# The asymptotic behaviour of parton distributions at small and large *x*

**DOI:** 10.1140/epjc/s10052-016-4240-4

**Published:** 2016-07-08

**Authors:** Richard D. Ball, Emanuele R. Nocera, Juan Rojo

**Affiliations:** 1 The Higgs Centre for Theoretical Physics, University of Edinburgh, JCMB, KB, Mayfield Rd., Edinburgh, EH9 3JZ Scotland; 2 Rudolf Peierls Centre for Theoretical Physics, University of Oxford, 1 Keble Road, Oxford, OX1 3NP UK

## Abstract

It has been argued from the earliest days of quantum chromodynamics that at asymptotically small values of *x* the parton distribution functions (PDFs) of the proton behave as $x^\alpha$, where the values of $\alpha$ can be deduced from Regge theory, while at asymptotically large values of *x* the PDFs behave as $(1-x)^\beta$, where the values of $\beta$ can be deduced from the Brodsky–Farrar quark counting rules. We critically examine these claims by extracting the exponents $\alpha$ and $\beta$ from various global fits of parton distributions, analysing their scale dependence, and comparing their values to the naive expectations. We find that for valence distributions both Regge theory and counting rules are confirmed, at least within uncertainties, while for sea quarks and gluons the results are less conclusive. We also compare results from various PDF fits for the structure function ratio $F_2^n/F_2^p$ at large *x*, and caution against unrealistic uncertainty estimates due to overconstrained parametrisations.

## Introduction

An accurate determination of parton distribution functions (PDFs) is an essential building block for the precision physics program at the large hadron collider (LHC) [1–5]. Given current limitations in the understanding of nonperturbative quantum chromodynamics (QCD), such a determination is not achievable from first principles. Instead, PDFs are determined in a global fit to hard-scattering experimental data [6–11], using perturbative QCD to combine information from different processes and scales. In such an analysis, the best-fit values of the input PDF parametrisation are obtained by comparing the PDF-dependent prediction of a suitable set of physical observables with their measured values, and then by minimising a figure of merit which quantifies the agreement between the two.

The parametrisation of the PDFs, $xf_i(x,Q_0^2)$, is set at an initial scale $Q_0^2$, and is then evolved to any other scale $Q^2$ via DGLAP equations [12–14]. The PDF parametrisation should be as general as possible, and in particular sufficiently smooth and flexible enough to accommodate all of the experimental data included in the fit without artificial bias. The kinematic constraint that $xf_i(x,Q_0^2)$ vanishes in the elastic limit $x\rightarrow 1$ should also be implicit in the parametrisation. Usually, the following *ansatz* is adopted

1 $$\begin{aligned} xf_i(x,Q_0^2) = A_{f_i}\, x^{a_{f_i}}\, (1-x)^{b_{f_i}}\, \mathscr {F} (x,\{c_{f_i}\}), \end{aligned}$$

where *x* is the parton momentum fraction and *i* denotes a given quark flavour (or flavour combination) or the gluon, and $\mathscr {F} (x,\{c_{f_i}\})$ is a smooth function which remains finite both when $x\rightarrow 0$ and $x\rightarrow 1$. The normalisation fractions $A_{f_i}$, the exponents $a_{f_i}$ and $b_{f_i}$, and the set of parameters $\{ c_{f_i}\}$ are then determined from the data. Some of the $A_{f_i}$ can be fixed in terms of the other fit parameters by means of the momentum and valence sum rules.

The original motivation for Eq. (1) was the theoretical expectation, based on nonperturbative QCD considerations, of a power-law behaviour of the PDFs at sufficiently small and large values of *x*. Specifically, Regge theory [15] predicts

2 $$\begin{aligned} xf_i(x,Q^2)\xrightarrow {x\rightarrow 0} x^{a_{f_i}}; \end{aligned}$$

while the Brodsky–Farrar quark counting rules [16] predict

3 $$\begin{aligned} xf_i(x,Q^2)\xrightarrow {x\rightarrow 1} (1-x)^{b_{f_i}}; \end{aligned}$$

see also Refs. [17, 18], and references therein. Both Regge theory and the counting rules provide numerical predictions for the values of the exponents $a_{f_i}$ and $b_{f_i}$. In Eq. (1), the small- and large-*x* power-law behaviours are matched at intermediate *x* values through the function $\mathscr {F}(x,\{c_{f_i}\})$. A number of different parametrisations have been used for this function so far, ranging from simple polynomials to more sophisticated Chebyshev [7, 19] and Bernstein [8] polynomials and multi-layer neural networks [20, 21].

It should be emphasised that Eqs. (2)–(3) *cannot* be derived using perturbative QCD, but rather require other more general considerations. For instance, counting rules can be derived from Bloom–Gilman duality [22] or using AdS/QCD methods in nonperturbative QCD [23].^1^ The use of Eqs. (2)–(3) in the input PDF parametrisation, Eq. (1), could therefore lead to theoretical bias. For instance, as we will discuss below, perturbative QCD calculations predict a logarithmic, rather than a power-like, growth of the PDFs at small *x*. Even if Eqs. (2)–(3) were a solid prediction from QCD (which they are not), they would not be particularly useful in the context of a global PDF analysis. First, it is unclear how small or large *x* should be in order for the power laws (2)–(3) to provide a reliably enough approximation of the underlying PDFs. Second, it is unclear at which values of $Q^2$ Regge theory and Brodsky–Farrar quark counting rules should apply exactly. This is a serious limitation, given the non-negligible PDF scale dependence around the input parametrisation scale $Q^2\simeq Q_0^2$. In principle, the optimal values of $Q^2$ should be chosen at the interface between perturbative and nonperturbative hadron dynamics, $Q^2\simeq Q_0^2 = Q^2_\mathrm{in}$. It has been shown [25] that $Q_\mathrm{in}^2\simeq 0.75$ GeV$^2$ by matching the high- and low-$Q^2$ behaviour of the strong coupling $\alpha _s(Q^2)$ as predicted respectively by its renormalisation group equation in the $\overline{\mathrm{MS}}$ scheme and its analytic form in the light-front holographic approach.

The aim of this study is to present a methodology to quantify the effective asymptotic behaviour of PDFs at small and large values of *x*, and then apply it to compare recent global fits with various perturbative and nonperturbative QCD predictions. The paper is organised as follows. In Sect. 2 we introduce a definition of the effective PDF exponents, and we use them to quantify for which ranges of *x* and $Q^2$, if any, PDFs exhibit a power-law behaviour of the form Eqs. (2)–(3). Once the asymptotic range has been determined, in Sect. 3 we investigate to which extent these exponents, as obtained from global PDF fits, are in agreement with the theoretical predictions of their values. In addition to Brodsky–Farrar quark counting rules, we will also compare the global fit predictions with other nonperturbative models of nucleon structure at large *x*. In principle, this comparison will allow us to discriminate among models, in the same way as was done for spin-dependent PDFs in Ref. [26].

## The effective exponents

In this paper we will compute the effective exponents $\alpha _{f_i}(x,Q^2)$ and $\beta _{f_i}(x,Q^2)$, which, when $Q^2=Q_0^2$, are asymptotically equal to the exponents $a_{f_i}$ and $b_{f_i}$ of the input PDF parametrisation Eq. (1). Specifically, we define

4 $$\begin{aligned}&\alpha _{f_i}(x,Q^2) \equiv \frac{\partial \ln [xf_i(x,Q^2)]}{\partial \ln x}, \nonumber \\&\beta _{f_i}(x,Q^2) \equiv \frac{\partial \ln [xf_i(x,Q^2)]}{\partial \ln (1-x)}\, , \end{aligned}$$

so that, at the input parametrisation scale $Q_0^2$,

5 $$\begin{aligned} \alpha _{f_i}(x,Q_0^2)= & {} a_{f_i} + x\left[ \frac{\mathrm{d}\ln [\mathscr {F}(x,\{c_{f_i}\})]}{\mathrm{d}x} - \frac{b_{f_i}}{1-x}\right] \xrightarrow {x\rightarrow 0} a_{f_i} \nonumber \\&+ \,O(x), \end{aligned}$$

and

6 $$\begin{aligned} \beta _{f_i}(x,Q_0^2)= & {} b_{f_i} - (1-x)\left[ \frac{\mathrm{d}\ln [\mathscr {F}(x,\{c_{f_i}\})]}{\mathrm{d}x} + \frac{a_{f_i}}{x}\right] \xrightarrow {x\rightarrow 1} b_{f_i} \nonumber \\&+\, O(1-x), \end{aligned}$$

since in both Eqs. (5) and (6) the term in square brackets is by construction of order one in the corresponding limit. Because subasymptotic terms of *O*(*x*) tend to zero very quickly at small *x*, and likewise subasymptotic terms of $O(1-x)$ tend to zero very quickly at large *x*, we expect that the definitions Eq. (4) $\alpha _{f_i}(x,Q^2)$ and $\beta _{f_i}(x,Q^2)$ can be used to accurately determine the asymptotic behaviour of any given PDF $xf_i(x,Q^2)$.

**Fig. 1:**
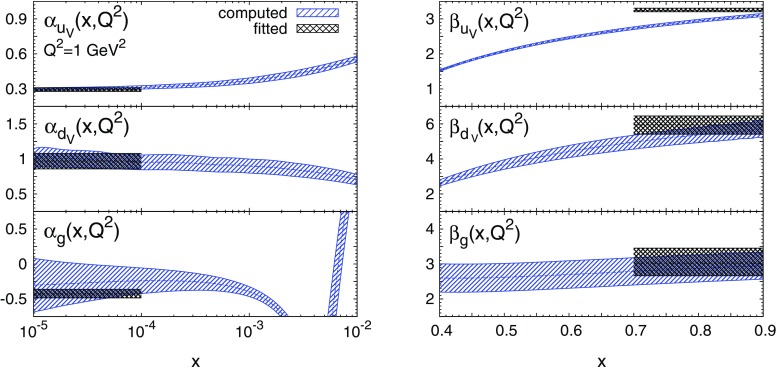
The effective exponents $\alpha _{f_i}(x,Q^2)$ (*left*) and $\beta _{f_i}(x,Q^2)$ (*right*) computed with Eq. (4). Results are shown at $Q^2=1$ GeV$^2$ for $u_V$, $d_V$ and *g* for the MSTW08 NLO PDF set. The shaded horizontal bands indicate the fitted values of the exponents $a_{f_i}$ (*left*) and $b_{f_1}$ (*right*) and their uncertainties. Numerical results at $x=10^{-5}$ and $x=0.9$ are collected in Table 1

In order to test this assertion, we have used Eq. (4) to compute the effective asymptotic exponents $\alpha _{f_i}(x,Q^2)$ and $\beta _{f_i}(x,Q^2)$ for the MSTW08 NLO PDF set [27] (see Appendix 1 for details). Results at $Q^2=1$ GeV$^2$, which coincides with the input parametrisation scale $Q_0^2$, are shown in Fig. 1 for the up valence quark, $f_i=u_V=u-\bar{u}$, the down valence quark, $f_i=d_V=d-\bar{d}$, and the gluon, $f_i=g$, PDFs. They are compared to the corresponding fitted exponents $a_{f_i}$ and $b_{f_i}$, to which they are expected to approach asymptotically. In Table 1 we show the numerical values computed respectively at $x=10^{-5}$ and $x=0.9$, and we again compare them with the corresponding fitted exponents $a_i$ and $b_i$.

From Fig. 1 and Table 1 it is clear that both $\alpha _{f_i}(x,Q^2)$ at $x=10^{-5}$ and $\beta _{f_i}(x,Q^2)$ at $x=0.9$ have converged to the fitted values of $a_{f_i}$ and $b_{f_i}$ within PDF uncertainties. In addition, by examining the *x* dependence of $\alpha _{f_i}(x,Q^2)$ and $\beta _{f_i}(x,Q^2)$, it is possible to identify the asymptotic regions in which they become roughly independent of *x*. Furthermore, since the definitions Eq. (4) may be applied at any value of $Q^2$, we may use them to study the $Q^2$ dependence of the effective exponents.

The definition of the PDF effective exponents, Eq. (4), is robust and we can therefore use it to compare the results of global fits among themselves and with different predictions from perturbative and nonperturbative QCD. We will focus on the up and down valence PDFs, $u_V=u-\bar{u}$ and $d_V=d-\bar{d}$, the total quark sea, $S=2(\bar{u} + \bar{d}) + s +\bar{s}$, and the gluon, *g*, from the NNPDF3.0 [6], MMHT14 [7], and CT14 [8] NNLO fits. We will also present some results from the ABM12 NNLO [9] and CJ15 NLO [11] sets. A detailed discussion of the similarities and differences between these PDF sets can be found in Refs. [2–4]; here we restrict ourselves to the information relevant for their small and large-*x* behaviour.

**Table 1:** The effective exponents $\alpha _{f_i}$ and $\beta _{f_i}$ at $Q^2=1$ GeV$^2$ and $x_a=10^{-5}$ and $x_b=0.9$ computed for the MSTW08 NLO PDF set with Eq. (4), compared to the corresponding fitted exponents $a_i$ and $b_i$

$f_{i}$	$\alpha _{f_i}(x_a,Q^2)$	$a_{f_i}$	$\beta _{f_i}(x_b,Q^2)$	$b_{f_i}$
$u_V$	$+0.29\pm 0.01$	$+0.291^{+0.019}_{-0.013}$	$+3.11\pm 0.04$	$+3.243^{+0.062}_{-0.039}$
$d_V$	$+1.02\pm 0.11$	$+0.968^{+0.110}_{-0.110}$	$+5.67\pm 0.47$	$+5.944^{+0.510}_{-0.530}$
*g*	$-0.30\pm 0.37$	$-0.428^{+0.066}_{-0.057}$	$+2.95\pm 0.39$	$+3.023^{+0.430}_{-0.360}$

- NNPDF3.0 PDFs are parametrised in the basis that diagonalises the DGLAP evolution equations [28]. The function $\mathscr {F}(x,\{c_{f_i}\})$ is a multi-layer feed-forward neural network (also known as *perceptron*). The power-law term $x^{a_{f_i}}(1-x)^{b_{f_i}}$ in Eq. (1) is treated as a preprocessing factor that optimises the minimisation process: the exponents $a_{f_i}$ and $b_{f_i}$ are chosen for each Monte Carlo replica at random in a given range determined iteratively.
- MMHT14 The PDFs parametrised are the valence distributions $u_V$ and $d_V$, the total sea *S*, the sea asymmetry $\Delta _S=\bar{d}-\bar{u}$, the total and valence strange distributions $s^+=s+\bar{s}$ and $s^-=s-\bar{s}$ and the gluon *g*. The function $\mathscr {F}(x,\{c_{f_i}\})$ is taken to be a linear combination of Chebyshev polynomials. The exponents $a_{f_i}$ and $b_{f_i}$ are fitted, except for $a_{s^+}=a_{S}$.
- CT14 The PDFs parametrised are the valence distributions $u_V$ and $d_V$, the sea quark distributions $\bar{u}$ and $\bar{d}$, the total strangeness $s^+$ and the gluon *g*. It is assumed that $s=\bar{s}$. The function $\mathscr {F}(x,\{c_{f_i}\})$ is a linear combination of Bernstein polynomials. The exponents $a_{f_i}$ and $b_{f_i}$ are parameters of the fit, but not all of them are free: specifically, it is assumed that $b_{u_V}=b_{d_V}$, so that as $x\rightarrow 1$ $u_V(x,Q_0^2)/d_V(x,Q_0^2)\rightarrow k$, with *k* a constant, and that as $x\rightarrow 0$ $\bar{u}(x,Q_0^2)/\bar{d}(x,Q_0^2)\rightarrow 1$, which requires $a_{\bar{u}}=a_{\bar{d}}$.
- ABM12 The PDFs parametrised are the valence distributions $u_V$ and $d_V$, the sea distributions $\bar{u}$ and *s*, the sea asymmetry $\Delta _S$ and the gluon *g*. It is assumed that $s=\bar{s}$. The function $\mathscr {F}(x,\{c_{f_i}\})$ has the form $x^{P_{f_i}(x)}$, where $P_{f_i}(x)$ is a function of *x*; for *s*, $\mathscr {F}(x,\{c_{f_i}\}=1$. The exponents $a_{f_i}$ and $b_{f_i}$ are parameters of the fit, except for the condition $a_{\Delta _S}=0.7$.
- CJ15 The PDFs parametrised are the valence distributions $u_V$ and $d_V$, the light antiquark sea, $\bar{u}+\bar{d}$, the light antiquark ratio $\bar{d}/\bar{u}$, the total strangeness $s^+$ and the gluon *g*. It is assumed that $s=\bar{s}$. The function $\mathscr {F}(x,\{ c_{f_i}\})$ is provided by the polynomial $(1+c_{f_i}^{(1)}\sqrt{x}+c_{f_i}^{(2)}x)$ for all the distributions except the light antiquark ratio and the total strangeness. Specifically, $\bar{d}/\bar{u}$ is parametrised with a simple polynomial which ensures that as $x\rightarrow 1$, $\bar{d}/\bar{u}\rightarrow 1$, while it is assumed that $s^+=\kappa (\bar{u}+\bar{d})$; $c_{f_i}^{(1)}$, $c_{f_i}^{(2)}$ and $\kappa$ are parameters of the fit. A small admixture of $u_V$ is added to $d_V$ so that as $x\rightarrow 1$ $d_V/u_V\rightarrow k$, with *k* a constant.

**Fig. 2:**
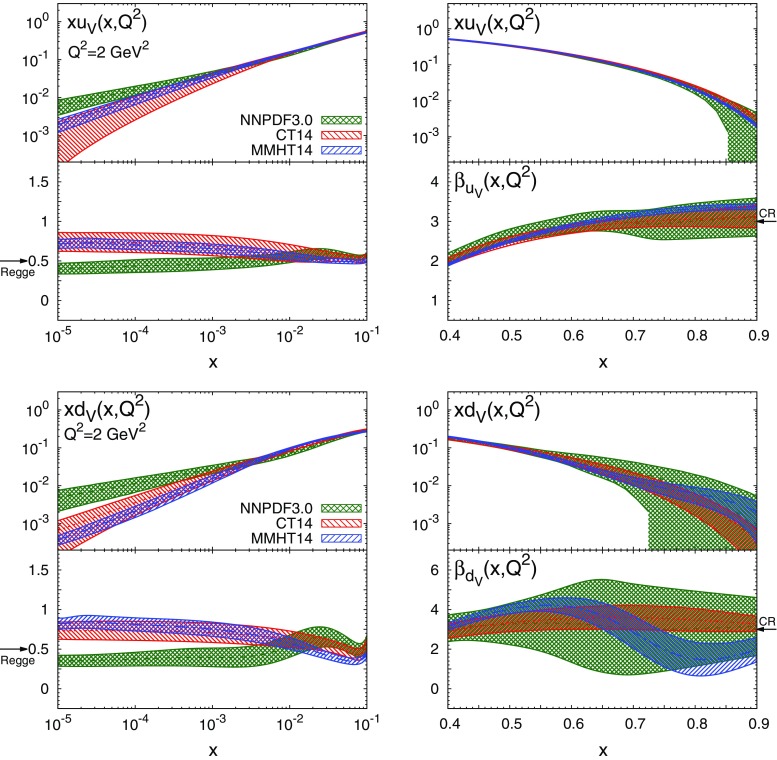
The effective exponents $\alpha _{f_i}(x,Q^2)$ (*left*) and $\beta _{f_i}(x,Q^2)$ (*right*), Eq. (4), for the up valence (*top*) and down valence (*bottom*) PDFs, as a function of *x* at $Q^2=2$ GeV$^2$, together with the corresponding PDFs. Results are shown for the NNPDF3.0, CT14 and MMHT14 NNLO PDF sets. The *arrows* indicate the prediction from Regge theory (Regge) and Brodsky–Farrar quark counting rules (CR)

Although the momentum distributions of strange and antistrange quarks are assumed to be identical in some of these PDF sets, it should be noted that a strange/antistrange asymmetry in the nucleon is predicted based on nonperturbative QCD models; see e.g. Ref. [29] and references therein. Strange and antistrange distributions may also be very different from each other in the polarised case, as it was shown in Ref. [29] based on a light-cone model of energetically favoured meson-baryon fluctuations applied to the $K^+\Lambda$. However, a study of a structured asymmetry in the momentum distributions of strange and antistrange quarks in a global QCD analysis is beyond the scope of this work, and has been addressed elsewhere [6, 7].

**Fig. 3:**
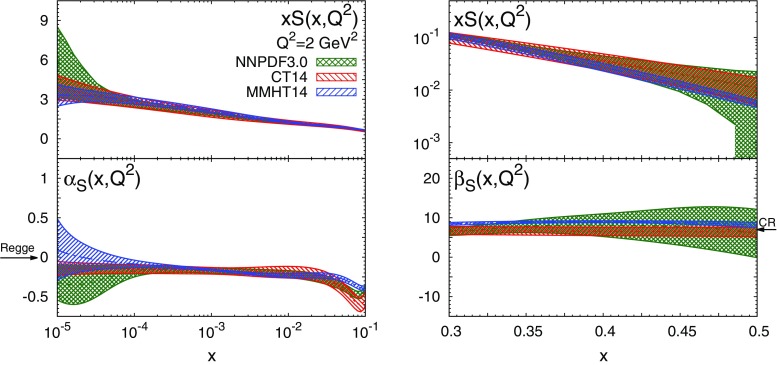
Same as Fig. 2 for the sea PDF $S(x,Q^2)$

**Fig. 4:**
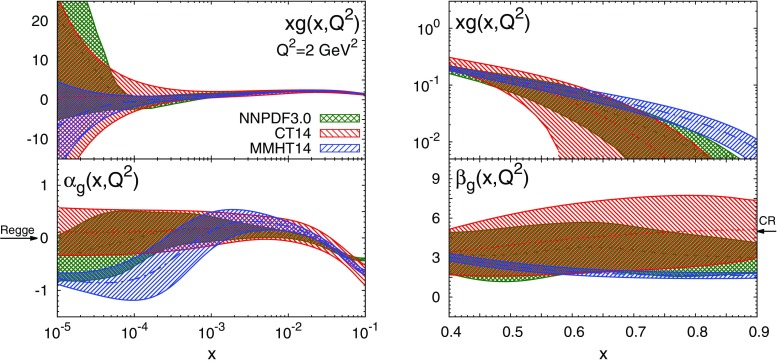
Same as Fig. 2 for the gluon PDF $g(x,Q^2)$

In Figs. 2, 3, 4 we compare both the PDFs and the corresponding effective exponents $\alpha _{f_i}(x,Q^2)$ and $\beta _{f_i}(x,Q^2)$ for the NNPDF3.0, MMHT14 and CT14 sets at $Q^2=2$ GeV$^2$. For NNPDF3.0, PDF uncertainties are computed as $68\%$ confidence level (CL) intervals, while for MMHT14 and CT14 sets we show the symmetric one-sigma Hessian uncertainties. In most cases it is possible to identify an asymptotic region where the effective exponents become approximately independent of *x*. The onset of this asymptotic regime depends on both the PDF flavour and on the PDF set. At small *x*, the asymptotic regime is reached at $x\lesssim 10^{-3}$ for $u_V$, $d_V$ and *S* irrespective of the PDF set considered. For the gluon, convergence is achieved at smaller values of *x*, $x\lesssim 10^{-5}$, at least for MMHT14 for which $\alpha _g(x,Q^2)$ has an oscillation in the region $10^{-4}\lesssim x\lesssim 10^{-3}$. Note that at $x\lesssim 10^{-4}$ PDFs are extrapolated into a region with very limited experimental information. This very small-*x* region can be probed at the LHC with forward charm [30, 31] and quarkonium production [32]. At large *x*, the asymptotic region is reached at $x\gtrsim 0.7$ in most cases. The exception is $\beta _{d_V}(x,Q^2)$ from MMHT14, which exhibits an oscillation in the region $0.6\lesssim x\lesssim 0.8$.

To the best of our knowledge, this is the first time that the onset of an asymptotic regime in the effective PDF exponents $\alpha _{f_i}(x,Q^2)$ and $\beta _{f_i}(x,Q^2)$ has been explicitly demonstrated. Remarkably, this onset takes place at *x* values close to the boundary between the data and extrapolation regions. Our results indicate that the three global PDF sets are broadly consistent among one other within uncertainties not only at the level of PDFs, but also at the level of their small- and large-*x* asymptotic behaviour. The main exceptions are $u_V$ and $d_V$ at small *x*, where the effective exponent of NNPDF3.0 is incompatible with those of CT14 and MMHT14. However, this is an extrapolation region where the Hessian approximation has some limitations and non-Gaussian effects are large: indeed, if we compute with NNPDF3.0 the one-sigma PDF interval as opposed to the $68\,\%$ CL, the three sets become consistent.

**Fig. 5:**
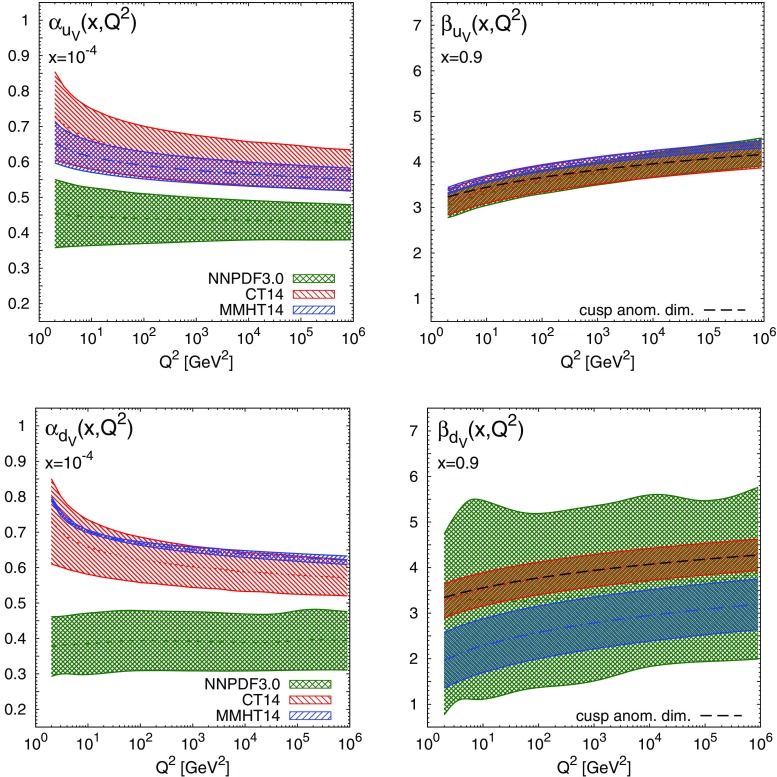
The effective exponents $\alpha _{f_i}(x,Q^2)$ (*left*) and $\beta _{f_i}(x,Q^2)$ (*right*), Eq. (4), for the up (*top*) and down valence (*bottom*) PDFs, as a function of $Q^2$ at $x=10^{-4}$ and $x=0.9$, respectively, for the NNPDF3.0, CT14 and MMHT14 NNLO sets. At large *x*, the perturbative QCD prediction Eq. (15) is also displayed for CT14

**Fig. 6:**
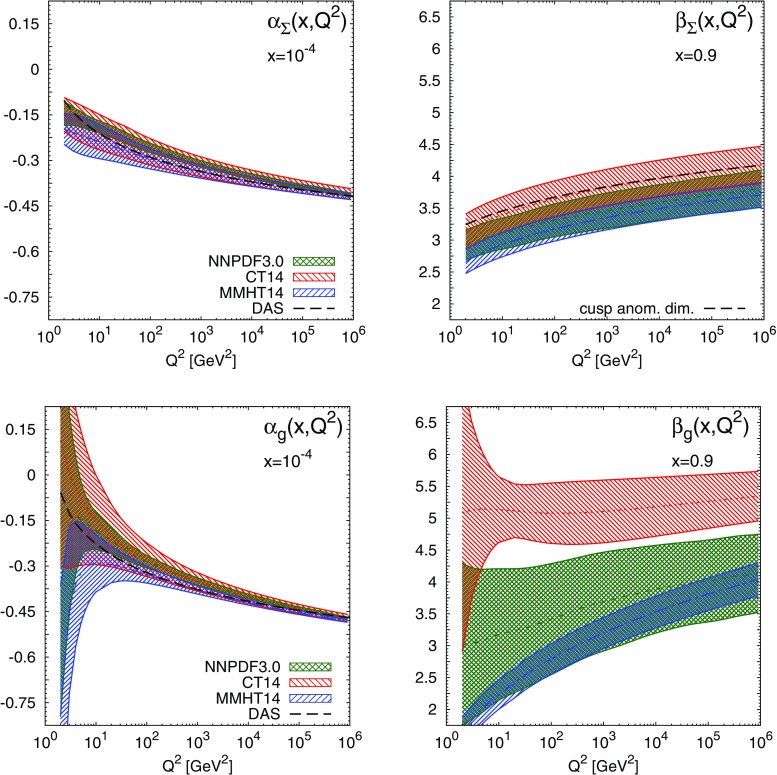
Same as Fig. 5 for the quark singlet $\Sigma$ and the gluon *g*. For $x=10^{-4}$ (*left plots*) we also show the DAS predictions, Eq. (10)

Before we compare our results to the expectations of Regge theory and the Brodsky–Farrar quark counting rules, we first examine the $Q^2$ dependence of the effective exponents. To this end, in Figs. 5, 6 we show the effective exponents $\alpha _{f_i}(x,Q^2)$ and $\beta _{f_i}(x,Q^2)$ as functions of $Q^2$ at fixed values of *x* in the asymptotic region: $x=10^{-4}$ and $x=0.9$, respectively. We show results for the valence distributions $u_V$ and $d_V$, the total quark singlet $\Sigma =\sum _{i=1}^{n_f} (q_i + \bar{q}_i)$ and the gluon. From these plots we can see that as $Q^2$ increases the effective exponents become less sensitive to $Q^2$ and tend to converge to a finite value asymptotically. This feature is broadly independent of *x* when *x* is sufficiently small or large, roughly $x\lesssim 10^{-3}$ and $x\gtrsim 0.9$. The only exception is again $\beta _{d_V}(x,Q^2)$ for MMHT14.

At small *x*, the $Q^2$ dependence of the effective exponents illustrates the transition from a low-$Q^2$ region, where PDFs are determined from nonperturbative dynamics, to a high-$Q^2$ region, where PDFs are dominated by perturbative QCD evolution. Indeed, as $x\rightarrow 0$ and $Q^2\rightarrow \infty$, PDFs can be solely determined by DGLAP equations [14, 33], provided that their behaviour is sufficiently soft at the input scale. In this limit, it is well known that PDFs exhibit a double asymptotic scaling (DAS) [34–37]. Specifically, as $x\rightarrow 0$ and $Q^2\rightarrow \infty$ the singlet sector grows as

7 $$\begin{aligned} x\Sigma (x,Q^2)\rightarrow & {} \mathcal{N}_\Sigma \frac{\gamma }{\rho }\frac{1}{\sqrt{4\pi \gamma \sigma }} e^{2\gamma \sigma - \delta \sigma /\rho },\nonumber \\ xg(x,Q^2)\rightarrow & {} \mathcal{N}_g\frac{1}{\sqrt{4\pi \gamma \sigma }}e^{2\gamma \sigma - \delta \sigma /\rho }, \end{aligned}$$

where we have defined

8 $$\begin{aligned} \gamma\equiv & {} \left( \frac{12}{\beta _0} \right) ^{1/2}\, ,\qquad \delta \equiv \left( 11 + \frac{2n_f}{27}\right) \Big /\beta _0, \nonumber \\ \beta _0= & {} 11 - \frac{2}{3}n_f\, , \end{aligned}$$

and the double scaling variables

9 $$\begin{aligned}&\sigma \equiv \left[ \ln \frac{x_0}{x} \ln \frac{\ln \left( Q^2/\Lambda ^2\right) }{\ln \left( Q_0^2/\Lambda ^2\right) } \right] ^{1/2},\nonumber \\&\rho \equiv \left[ \frac{\ln \left( x_0/x\right) }{\ln \left( \ln \left( Q^2/\Lambda ^2\right) /\ln \left( Q_0^2/\Lambda ^2\right) \right) } \right] ^{1/2} \,\text{. } \end{aligned}$$

The parameters $x_0$ and $Q_0^2$ define the formal boundaries of the asymptotic region, $\mathcal{{N}}_\Sigma$ and $\mathcal{{N}}_g$ are normalisation constants, and $n_f$ is the number of active flavours. Using the asymptotic form Eq. (7) in the definition of the effective exponents Eq. (4) then gives us a perturbative prediction for the small-*x* exponents $\alpha _{\Sigma }$ and $\alpha _g$: at large $\sigma$ but fixed $\rho$ one has

10 $$\begin{aligned} \alpha _{\Sigma }(x,Q^2) \rightarrow -\frac{\gamma }{\rho }+\frac{3}{4\sigma \rho },\quad \alpha _{g}(x,Q^2) \rightarrow -\frac{\gamma }{\rho }+ \frac{1}{4\sigma \rho }. \end{aligned}$$

Note that both $\alpha _{\Sigma }(x,Q^2)$ and $\alpha _g(x,Q^2)$ converge asymptotically to the same value $-\gamma /\rho$, as expected since the QCD evolution of the gluon distribution seeds the evolution of the quark singlet distribution. The DAS results Eq. (10), which are a generic prediction of perturbative QCD, are displayed in Fig. 6, where we have used $x_0=0.1$, $Q_0^2=1$ GeV$^2$, $n_f=5$ and $\Lambda ^{(n_f=5)}=0.220$ GeV. The agreement between the expectation from DAS and results from the global fits is excellent at $Q^2\gtrsim 10$ GeV$^2$ for both the quark singlet and the gluon.

At large *x*, the $Q^2$ dependence of the effective exponents can also be determined from general perturbative QCD considerations, following directly from the universality of the cusp quark anomalous dimension in the $\overline{\mathrm{MS}}$ scheme [38, 39]. Specifically, it can be shown, either by analysing Wilson lines [38], or by using standard results for the exponentiation of soft logarithms in the quark-initiated bare cross sections [39], that the quark anomalous dimension at large *N* takes the universal form

11 $$\begin{aligned} \gamma _q(N,\alpha _s(q^2))\sim - c(\alpha _s(q^2))\ln N + d(\alpha _s(q^2))+O(1/N), \end{aligned}$$

where $c(\alpha _s(q^2))$ and $d(\alpha _s(q^2))$ can be computed perturbatively: for example at NLO

12 $$\begin{aligned} c(\alpha _s(q^2)) = \frac{\alpha _s(q^2)}{2\pi }c_1 + \left( \frac{\alpha _s(q^2)}{2\pi }\right) ^2 c_2 + O(\alpha _s^3), \end{aligned}$$

**Table 2:** The values of the small-*x* effective exponent $\alpha _{f_i}(x_a,Q^2)$ computed at $Q^2=2$ GeV$^2$ and $Q^2=10$ GeV$^2$ at $x_a=10^{-4}$, compared to the values of $a_{f_i}$ predicted by Regge theory (and resummation of double logarithms). For the quark sea *S* and the gluon *g* we indicate the prediction of the soft Pomeron (and the NLLx perturbative result)

$f_i$	$Q^2$	$\alpha _{f_i}(x_a,Q^2)$					$a_{f_i}$
	(GeV$^2$)	NNPDF3.0	CT14	MMHT14	ABM12	CJ15	
$u_V$	2.0	$+0.48\pm 0.11$	$+0.72\pm 0.12$	$+0.65\pm 0.06$	$+0.76\pm 0.07$	$+0.61\pm 0.01$	$+0.5$
	10.0	$+0.46\pm 0.09$	$+0.66\pm 0.09$	$+0.61\pm 0.04$	$+0.70\pm 0.04$	$+0.60\pm 0.01$	(0.63)
$d_V$	2.0	$+0.41\pm 0.11$	$+0.73\pm 0.12$	$+0.79\pm 0.06$	$+1.39\pm 0.10$	$+1.11\pm 0.03$	$+0.5$
	10.0	$+0.41\pm 0.11$	$+0.66\pm 0.07$	$+0.70\pm 0.04$	$+0.91\pm 0.08$	$+0.95\pm 0.05$	(0.63)
*S*	2.0	$-0.14\pm 0.06$	$-0.15\pm 0.05$	$-0.09\pm 0.04$	$-0.16\pm 0.02$	$-0.18\pm 0.03$	$-0.08$
	10.0	$-0.18\pm 0.04$	$-0.20\pm 0.05$	$-0.15\pm 0.04$	$-0.19\pm 0.01$	$-0.14\pm 0.02$	($-0.2$)
*g*	2.0	$-0.16\pm 0.63$	$+0.06\pm 0.31$	$-0.79\pm 0.43$	$+0.18\pm 0.10$	$+0.08\pm 0.03$	$-0.08$
	10.0	$-0.20\pm 0.46$	$-0.15\pm 0.15$	$-0.29\pm 0.09$	$-0.15\pm 0.01$	$-0.14\pm 0.01$	($-0.2$)

**Table 3:** Same as Table 2 for the large-*x* effective exponent $\beta _{f_i}(x_b,Q^2)$ at $x_b=0.9$ (for $u_V$, $d_V$ and *g*) and $x_b=0.5$ (for *S*). The values of the exponent $b_{f_i}$ predicted by Brodsky–Farrar quark counting rules are also shown

$f_i$	$Q^2$	$\beta _{f_i}(x_b,Q^2)$					$b_{f_i}$
	(GeV$^2$)	NNPDF3.0	CT14	MMHT14	ABM12	CJ15	
$u_V$	2.0	$+2.94\pm 0.52$	$+3.11\pm 0.28$	$+3.37\pm 0.07$	$+3.38\pm 0.06$	$+3.50\pm 0.01$	${\sim }3$
	10.0	$+3.30\pm 0.69$	$+3.38\pm 0.29$	$+3.62\pm 0.07$	$+3.61\pm 0.05$	$+3.78\pm 0.01$	
$d_V$	2.0	$+3.03\pm 1.96$	$+3.27\pm 0.37$	$+2.05\pm 0.59$	$+4.72\pm 0.43$	$+3.42\pm 0.06$	${\sim }3$
	10.0	$+3.23\pm 1.88$	$+3.52\pm 0.36$	$+2.29\pm 0.59$	$+4.92\pm 0.42$	$+3.68\pm 0.05$	
*S*	2.0	$+6.86 \pm 7.25$	$+6.41 \pm 1.22$	$+8.19 \pm 0.68$	$+8.16 \pm 0.38$	$+7.73 \pm 0.18$	${\sim }7$
	10.0	$+6.76 \pm 6.71$	$+6.91 \pm 1.14$	$+6.83 \pm 0.88$	$+8.51 \pm 0.38$	$+8.15 \pm 0.18$	
*g*	2.0	$+2.95\pm 1.25$	$+5.08\pm 2.18$	$+1.65\pm 0.23$	$+4.18\pm 0.06$	$+6.11\pm 0.33$	${\sim }5$
	10.0	$+3.25\pm 0.98$	$+5.13\pm 0.51$	$+2.24\pm 0.23$	$+4.44\pm 0.06$	$+4.91\pm 0.33$	

with coefficients [40]

13 $$\begin{aligned} c_1 = \frac{8}{3},\quad c_2 = 4\left( \frac{67}{9} - 2\zeta _2\right) - \frac{40}{27} n_f. \end{aligned}$$

It follows [39] that, if $xf_{q}(x,Q_0^2)\sim (1-x)^{b(Q_0^2)}$ as $x\rightarrow 1$ at a scale $Q_0^2$, with *q* either the quark singlet, $\Sigma$, or one of the quark valence distributions, $u_V$ or $d_V$, then this asymptotic behaviour persists at higher scales $Q^2$ with

14 $$\begin{aligned} b(Q^2) = b(Q_0^2)+\int _{Q_0^2}^{Q^2}\frac{dq^2}{q^2}c(\alpha _s(q^2)). \end{aligned}$$

Given our definition Eq. (4) and the asymptotic behaviour Eq. (6) at large *x*, as $x\rightarrow 1$ one has

15 $$\begin{aligned} \beta _{f_i}(x,Q^2) = \beta _{f_i}(x,Q_0^2)+\int _{Q_0^2}^{Q^2}\frac{dq^2}{q^2}c(\alpha _s(q^2)). \end{aligned}$$

The behaviour predicted by Eq. (15) is displayed for $u_V$ and $d_V$ in Fig. 5, and for $\Sigma$ in Fig. 6. Note that Eq. (15) only determines the shape of the curve, not its overall normalisation; for definiteness we fix the value of $\beta (x,Q_0^2)$ in Eq. (15) to match the central values obtained from CT14 at $Q^2=10^6$ GeV$^2$. The agreement between Eq. (15) and the $Q^2$ dependence of the large-*x* effective exponents derived from the PDF fit is excellent. A slight deterioration only appears at small values of $Q^2$ due to missing higher order corrections. Similar conclusions can be derived for other PDF sets when the value of $\beta _{f_i}(x,Q_0^2)$ in Eq. (15) is assigned consistently.

## Comparison with nonperturbative predictions

We now discuss how our findings compare with the expectations from Regge theory and the Brodsky–Farrar quark counting rules. In Tables 2, 3 we show the values of the effective exponents for the NNPDF3.0, CT14, MMHT14, ABM12 and CJ15 PDF sets, computed at $x_a=10^{-4}$ and $x_b=0.9$ ($x_b=0.5$ for *S*) at $Q^2=2$ GeV$^2$ and $Q^2=10$ GeV$^2$. We also include the values predicted by Regge theory and the Brodsky–Farrar quark counting rules.

At small *x*, Regge theory predicts $xf_i\sim x^{a_{f_i}}$ with $a_{f_i}$ a $Q^2$-independent exponent, related to the intercept of the corresponding Regge trajectory. For valence quark distributions, a value of $a_{u_V}=a_{d_V}\simeq +0.5$ is derived from the non-singlet Regge trajectory intercept $1-\alpha _R(0)$. Perturbative calculations which resum the double logarithms of *x* give a similar value $a_{u_V}=a_{d_V}\simeq +0.63$ [41, 42]. For the gluon distribution, a value of $a_g$ close to the singlet Pomeron trajectory $1-\alpha _P(0)$ is expected; the conventional Regge exchange is that of the soft Pomeron [43] (for a formulation of the parton picture without recourse to perturbation theory see also Ref. [44]), leading to $a_g\simeq -0.08$. Attempts to compute the Pomeron intercept perturbatively by solution of the fixed coupling LLx BFKL equation [45–48] give $a_g\simeq -0.5$. However, this result is destabilised by NLLx corrections [49]. When running coupling effects are taken into account, the perturbative expansion is stabilised [50–54], and the NLLx perturbative prediction becomes $a_g\simeq -0.2$. For the total sea distribution, the value of $a_S$ should be similar for large enough $Q^2$ to $a_g$, due to the dominance of the process $g\rightarrow q\bar{q}$ in the evolution of sea quarks.

**Fig. 7:**
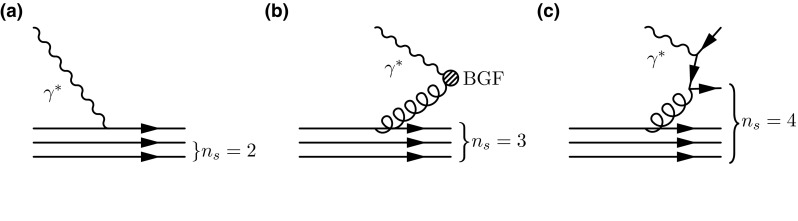
The number of *spectator* partons in a proton consisting of three quarks, whether a valence quark (**a**), a gluon (**b**) or a sea quark (**c**) is struck by a virtual photon $\gamma ^*$ in deep-inelastic scattering

In comparing these expectations with the results from PDF fits, we need to choose a scale. Regge predictions are expected to hold only at low scales. For $\alpha _{u_V}(x,Q^2)$ and $\alpha _{d_V}(x,Q^2)$ this is not too much of a problem, since the scale dependence of non-singlet distributions is quite weak (see Fig. 5). The values extracted from NNPDF3.0 are accordingly in good agreement with Regge expectations; those from the other global PDF fits are generally a little high (see Table 2). On the other hand, for $\alpha _S(x,Q^2)\simeq \alpha _\Sigma (x,Q^2)$ and $\alpha _g(x,Q^2)$, the scale dependence is rather strong (see Fig. 6), due to the double scaling behaviour. Making the comparison at low scales, we see reasonable agreement for the sea quarks with the Pomeron prediction, and also with the NLLx perturbative prediction. Uncertainties for the gluon intercept are inevitably large, so here the agreement is only qualitative. Note that for ABM12 and CJ15 the uncertainties are often substantially underestimated due to parametrisation constraints in the extrapolation region.

At large *x*, the Brodsky–Farrar quark counting rules predict that $xf_i\sim (1-x)^{2n_s-1}$, where $n_s$ is the minimum number of *spectator* partons. These are defined to be the partons that are not struck in the hard-scattering process, since it is assumed that, in the limit $x\rightarrow 1$, there can be no momentum left for any of the partons other than the struck parton. In a proton made of three quarks, one has for a valence quark, $n_s=2$ and thus $b_{u_V}=b_{d_V}=3$; for a gluon, $n_s=3$ and $b_g=5$; for a sea quark, $n_s=4$ and $b_S=7$; see Fig. 7. Note that the values of the exponents predicted by Brodsky–Farrar quark counting rules are different if the polarisation of the quark with respect to the polarisation of the parent hadron is retained [55]. This also affects the difference between up and down distributions. A detailed comparison between PDFs and quark counting rules in the polarised case was presented in Ref. [26]. Again it is unclear from the quark model argument at which scale these predictions are supposed to apply, but again we are fortunate that the scale dependence of large-*x* PDFs is reasonably moderate (see Figs. 5, 6), and it is reasonable to make the comparison at a low scale [25].

The predictions $b_{u_V}(x,Q^2)$ and $b_{d_V}(x,Q^2)$ for the valence distributions are then in broad agreement with the effective exponents determined from most of the global PDF fits, though some deviations from Brodsky–Farrar quark counting rule expectations are observed for the MMHT14 down valence quarks: this seems to be a result of the oscillation noted already in Fig. 2. For the quark sea and the gluon, the success is again rather mixed, and only CT14 seems to provide results which agree with the prediction; for NNPDF3.0 the uncertainties on the quark sea are too large for the extraction to be meaningful, while the result for the gluon is a little low; for MMHT14 the result for the gluon is far too low, with a substantially underestimated uncertainty.

In addition to the Brodsky–Farrar quark counting rules, the behaviour of PDFs at large *x* has been predicted by several nonperturbative models of nucleon structure (see *e.g.* [56, 57] and references therein). In many cases, these provide expectations for the ratio of *u* to *d* valence distributions in the proton, $d_V/u_V$, and of neutron to proton structure functions, $F_2^n/F_2^p$. These ratios are particularly interesting because while all PDFs vanish at $x=1$, their ratio does not necessarily do so, and thus it is a useful discriminator among models of nucleon structure.

**Fig. 8:**
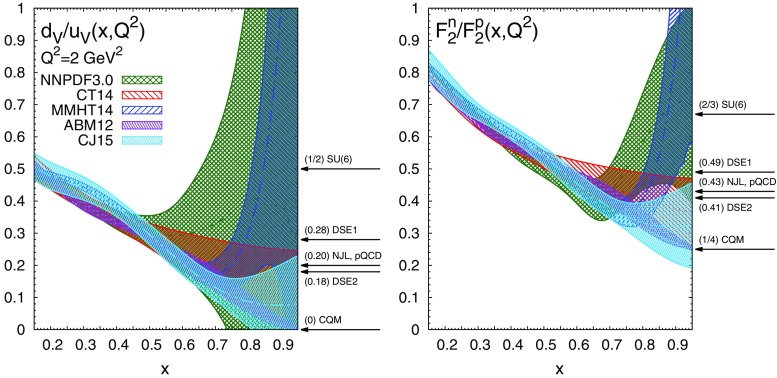
The ratios $d_V/u_V$ (*left*) and $F_2^n/F_2^p$ (*right*) at $Q^2=2$ GeV$^2$ among various PDF sets, compared with the predictions of different nonperturbative models of nucleon structure

In the parametrisation Eq. (1), $d_V/u_V\sim (1-x)^{b_{d_V}-b_{u_V}}$ as $x\rightarrow 1$, so if $b_{u_V}=b_{d_V}$, as predicted by the counting rules, then $d_V/u_V\rightarrow k$, with *k* some constant. Indeed it is the constant *k* that many of the models try to predict. Moreover, as noted above, both CT14 and CJ15 assume $b_{u_V}=b_{d_V}$ in their fits. However, while one may expect $b_{u_V}\simeq b_{d_V}$ because of isospin symmetry, it is also reasonable to expect that exact equality will be broken by isospin breaking or electromagnetic effects. The sign of these effects is crucial: if $b_{u_V}>b_{d_V}$ then $d_V/u_V$ will become infinite as $x\rightarrow 1$, while if $b_{u_V}<b_{d_V}$, as $x\rightarrow 1$ $d_V/u_V\rightarrow 0$. These two possibilities result in naive limits on the ratio $F_2^n/F_2^p$: if the sea quarks can be ignored at large *x*, then $d_V\gg u_V$, $F_2^n/F_2^p\rightarrow 4$, while for $d_V\ll u_V$ $F_2^n/F_2^p\rightarrow 1/4$, giving for $x\rightarrow 1$ the Nachtmann limits [58]

16 $$\begin{aligned} \frac{1}{4}\le \frac{F_2^n}{F_2^p}\le 4\, . \end{aligned}$$

To address these issues empirically, in Fig. 8 we compare the ratios $d_V/u_V(x,Q^2)$ and $F_2^n(x,Q^2)/F_2^p(x,Q^2)$ at $Q^2=2$ GeV$^2$ as predicted by the various PDF sets. The neutron and proton structure functions $F_2^n(x,Q^2)$ and $F_2^p(x,Q^2)$ have been computed at NNLO accuracy with APFEL [59] using the FONLL-C general-mass scheme [60]. The arrows on the right hand side of each panel indicate the expectations from a representative set of nonperturbative models of nucleon structure: $\mathrm{\mathtt SU(6)}$ [61] describes constituent quarks in the nucleon by $\mathrm{SU(6)}$ wave functions; CQM [62, 63] is the relativistic Constituent Quark Model in which a $\mathrm{SU(6)}$ symmetry breaking is assumed via a color hyperfine interaction between quarks; NJL [64] is a modified Nambu–Jona-Lasinio model in which confinement is simulated by eliminating unphysical thresholds for nucleon decay; pQCD [65] stands for a coloured quark and vector gluon model supplemented with leading order perturbative QCD; DSE1 and DSE2 [66] are two scenarios based on Dyson–Schwinger equations.

From Fig. 8, we see that in the region in which the valence quarks are constrained by experimental data, i.e. $x\lesssim 0.5$, the predictions for both ratios from all the PDF sets are in reasonable agreement with each other within uncertainties, as might be expected. For $x\gtrsim 0.5$, the mutual consistency of PDF sets deteriorates rapidly, and a wide range of different behaviours is observed. This is a consequence of the reduced experimental information in this region: different PDF collaborations extrapolate to large *x* using different assumptions. For those sets with very weak assumptions on the PDF behaviour at large *x*, namely NNPDF3.0 and MMHT14, the uncertainties on the ratios expand rapidly, and at very large *x* there is no predictive power at all. For the two sets which assume that $d_V/u_V\rightarrow k$ at large *x*, namely CT14 and CJ15, uncertainties are inevitably much reduced and a value of *k* is predicted. ABM12 is different again, in that they find as a result of their fit that $b_{d_V}>b_{u_V}$ at more than two standard deviations (see Table 3), so that $d_V/u_V\rightarrow 0$ as $x\rightarrow 1$, and an unrealistically small uncertainty band in a region where there are actually no data.

It follows that all the various model predictions displayed in Fig. 8 are compatible with the NNPDF3.0 and MMHT14 predictions, while ABM12 confirms the Chiral Quark Model but appears to rule out all the others. The CT14 and CJ15 sets favour values of *k* in the region $0\lesssim k \lesssim 0.25$, thus disfavouring the *SU*(6) prediction but unable to discriminate between the others. The preference for smaller values of *k* results in effect from a linear extrapolation of the downwards trend in the data region $x\lesssim 0.5$. Not all the predictions respect the Nachtmann bound, Eq. (16).

## Conclusions and outlook

In summary, in this work we have introduced a novel methodology to determine quantitatively the effective asymptotic behaviour of parton distributions, valid for any value of *x* and $Q^2$. For the first time, we have unambiguously identified the ranges in *x* and $Q^2$ where the asymptotic regime sets in, allowing us to compare in detail perturbative and nonperturbative QCD predictions at large and small *x* with the results of modern global PDF fits.

Concerning the small-*x* region, we have found broad agreement between the results from PDF fits and the predictions from Regge theory for the behaviour of the valence quark distributions. For the singlet and gluon distributions, the agreement with Regge predictions is still only qualitative, due in part to the substantial scale dependence, as well as the limited experimental information available in that region. On the other hand, the perturbative QCD double asymptotic scaling predictions are in excellent agreement with the results of PDFs fits over a wide range of $Q^2$.

Concerning the large-*x* region, we have found that the predictions of the Brodsky–Farrar counting rules for the behaviour of the valence quark distributions are in broad agreement with the global fit results, within PDF uncertainties. For the sea and gluon distributions uncertainties are much larger, and the agreement is only qualitative. The scale dependence of the effective exponents based on global PDF fits is in excellent agreement with the perturbative QCD expectation from the cusp anomalous dimension in a wide range of $Q^2$. We have also compared the ratios $d_V(x,Q^2)/u_V(x,Q^2)$ and $F_2^n(x,Q^2)/F_2^p(x,Q^2)$ among PDF fits and with nonperturbative models of nucleon structure, but found that the interpretation of this comparison depends significantly on the assumptions built into the PDF parametrisation, to the extent that it is impossible at present to draw any firm conclusions.

We therefore conclude that, while the ancient wisdom of Regge theory and the Brodsky–Farrar counting rules seems to have some degree of truth, particularly in the valence quark sector, they are no substitute for the precise empirical PDF determinations provided by global analysis, and when used as constraints may lead to unrealistically accurate predictions in kinematic regions where there is no experimental data. Global PDF fits will always be hampered to some extent by the lack of data to constrain PDFs in extrapolation regions, and new measurements from the LHC and other facilities, such as JLab, are required to shed more light on the asymptotic behaviour of parton distributions at small and large *x*. The methodology presented in this work should find applications in future comparisons between different global PDF fits, and between PDF fits and nonperturbative models of nucleon structure.

## Figures and Tables

**Fig. 9:**
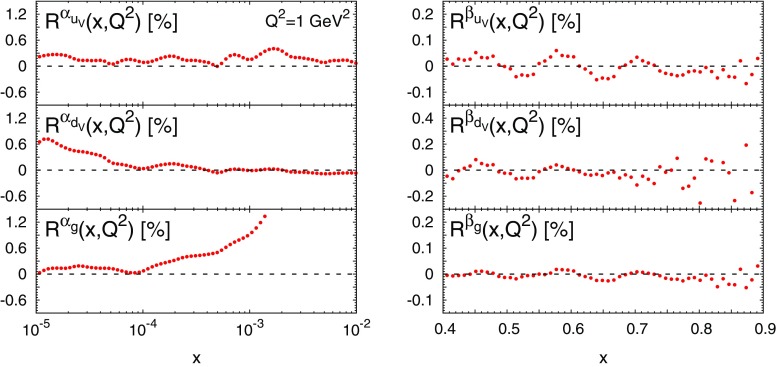
The percentage difference $R(x,Q^2)$, Eq. (17), between the numeric and analytic evaluation of the effective exponents $\alpha _{f_i}$ (*left*) and $\beta _{f_i}$ (*right*) for the MSTW08 NLO set at $Q^2=1$ GeV$^2$

## Appendix: Numerical determination of the effective exponents

The accurate evaluation of the effective exponents $\alpha _{f_i}(x,Q^2)$ and $\beta _{f_i}(x,Q^2)$ through Eq. (4) is pivotal in our study. An analytic evaluation of Eq. (4) starting from the explicit PDF parametrisation in Eq. (1), though straightforward, has two main limitations. First, Eq. (1) holds only at the initial parametrisation scale $Q_0^2$; the form of Eq. (1) is rapidly washed out by DGLAP evolution, hence it cannot be used for the analytic computation of the effective exponents at $Q^2 > Q_0^2$. Second, even at $Q^2=Q_0^2$, only the best-fit parameters for the central PDF are provided, and, moreover, for some PDF fits not even a simple analytical parametrisation is used.

To overcome these difficulties, in this work we evaluate Eq. (4) numerically. To this purpose, PDFs in a suitable numerical format and an algorithm for the numerical computation of the logarithmic derivative of the PDF in Eq. (4) are necessary. The first requirement is fulfilled by LHAPDF6 [67], while the second is more delicate. The standard methods used to evaluate numerical derivatives, such as those based on a finite difference approximation or on a polynomial approximation of the function to be derived, see e.g. Sects. 5.7–5.9 in Ref. [68], are found to lead to unstable results. The reason is that PDFs available through LHAPDF6 are tabulated on a grid in $(x,Q^2)$; the values of the PDFs off a grid node are then obtained by a cubic spline interpolation. This interpolation induces small fluctuations of the PDFs with respect to their *true* value, in particular in the small and large-*x* regions, where the grid tabulations are less dense. Such fluctuations are enhanced when the numerical derivative of the PDF is computed, especially if the value of the PDF is very small, thus spoiling the evaluation of Eq. (4).

We overcome this problem and perform the numerical derivative in Eq. (4) by means of a Savitzky–Golay smoothing filter [69]. The idea is the following. Assuming that a function *g*(*x*) is tabulated at $n+1$ equally spaced intervals, $g_i\equiv g(x_i)$, with $x_i=x_0+i\Delta$ for some constant sample spacing $\Delta =(x_n-x_0)/n$ and $i=-n/2,\ldots ,-2,-1,0,1,2,\ldots ,n/2$, the filter performs a least-squares fit with a polynomial of some degree *m* at each point, using an additional number $n_L$ of points to the left and some number $n_R$ of points to the right of each desired *x* value. The estimated derivative is then the derivative of the resulting fitted polynomial. The values of the parameters $x_0$, $x_n$, *n*, $n_L$, $n_R$ and *m* are optimised for each flavour and PDF set, so that residual numerical instabilities are minimised.

The robustness of our numerical procedure can be validated by comparing it with an analytic evaluation of Eq. (4). For instance, we consider the MSTW08 NLO PDF set at $Q^2=1$ GeV$^2$, and compute the central value of the effective exponents $\alpha _{f_i}(x,Q^2)$ and $\beta _{f_i}(x,Q^2)$ both analytically and numerically. The relative difference between the two computations, defined as

17 $$\begin{aligned}&R^{\alpha _{f_i}}(x,Q^2) = \frac{\alpha _{f_i}^\mathrm{(num)}(x,Q^2) - \alpha _{f_i}^\mathrm{(ana)}(x,Q^2)}{\alpha _{f_i}^\mathrm{(ana)}(x,Q^2)} \nonumber \\&R^{\beta _{f_i}}(x,Q^2) = \frac{\beta _{f_i}^\mathrm{(num)}(x,Q^2) - \beta _{f_i}^\mathrm{(ana)}(x,Q^2)}{\beta _{f_i}^\mathrm{(ana)}(x,Q^2)}, \end{aligned}$$

is displayed in Fig. 9 for the $u_V$, $d_V$ and *g* PDFs. The agreement between the analytic (ana) and numeric (num) computation is excellent: relative differences are at the permille level, with the only exception of $\alpha _g(x,Q^2)$ around $x\sim 10^{-3}$, where the input gluon PDF has a node.

## Footnotes

1 It has been proved that counting rules are rigorous predictions of QCD, modulo calculable logarithmic corrections from the behaviour of the hadronic wave function at short distances, in the case of large momentum transfer exclusive processes [22, 24].
